# Diversity analysis of endohyphal bacteria in oil-producing fungi inhabiting arid environments

**DOI:** 10.3389/fmicb.2025.1712713

**Published:** 2026-01-06

**Authors:** Yanxia Liang, Yulian Feng, Qiong Jia, Jing Zhu, Shengting Guo, Qiyong Tang, Yonghong Fan, Zhidong Zhang

**Affiliations:** 1 College of Life Science and Technology, Xinjiang University, Urumqi, China; 2 Xinjiang Key Laboratory of Special Environmental Microbiology/National Collection of Microbial Resource for Fertilizer (Xinjiang), Institute of Microbiology, Xinjiang Academy of Agricultural Sciences, Urumqi, China; 3 College of Food Sciences and Pharmacy, Xinjiang Agricultural University, Urumqi, China

**Keywords:** arid environments, diversity, endohyphal bacteria, high-throughput sequencing, oil-producing fungi

## Abstract

**Introduction:**

Endohyphal bacteria are microorganisms that inhabit fungal hyphae or reproductive structures, which influence fungal physiology and contribute to broader ecosystem processes. However, current knowledge regarding endohyphal bacteria associated with oil-producing fungi in arid environments remains scarce and warrants further investigation.

**Method:**

Oil-producing fungi were isolated and screened from arid soil samples collected in Toksun County, Xinjiang, China. A preliminary assessment of the presence of endohyphal bacteria within these fungi was conducted using SYTO 9 green fluorescent staining. High-throughput sequencing was employed to analyze the distribution patterns and community composition of the endohyphal bacteria.

**Results:**

Endohyphal bacteria were detected in 16 fungal strains, which constituted 61.5% of the total 26 oil-producing strains obtained. High-throughput sequencing analysis identified 63 amplicon sequence variants (ASVs) belonging to 6 phyla and 35 genera, with Proteobacteria representing the dominant phylum. Most fungi contained multiple endohyphal bacterial taxa and exhibited pronounced interspecific variation in community composition. Functional prediction analysis indicated a significant enrichment of pathways related to metabolism, environmental information processing, and genetic information processing in the endohyphal bacteria associated with distinct fungal hosts.

**Discussion:**

Oil-producing fungi may establish interactive systems through symbiotic associations with diverse endohyphal bacteria. These symbiotic interactions may promote lipid accumulation and enhance ecological adaptability in oil-producing fungi, potentially mediated by the metabolic synergy and functional complementarity described above. In conclusion, this study provides a preliminary characterization of the diversity and community structure of endohyphal bacteria associated with oil-producing fungi in arid environments, establishing a basis for future investigations into their functional interactions.

## 1 Introduction

Endohyphal bacteria (EHB) are microorganisms that reside within the vegetative hyphae or reproductive structures of fungi (Liu et al., 2019). They establish mutually beneficial symbiotic relationships with their fungal host and play crucial roles in microbial adaptation and evolution (Hoffman et al., 2013). Recent studies have revealed the widespread occurrence of endohyphal bacteria across major fungal phyla, including Ascomycota (Hoffman and Arnold, 2010), Basidiomycota (Hassan et al., 2022), and Mucoromycota (Alessandro et al., 2023). Robinson et al. (2021) reported diverse endohyphal bacterial communities across 617 fungal strains spanning multiple phyla. This intimate symbiotic relationship is characterized by bidirectional exchange of materials and functional complementarity: fungi provide stable habitats and nutrient supply for endohyphal bacteria (Anca et al., 2009), whereas these bacteria, in turn, modulate fungal growth and metabolism (Arendt, 2015; Shaffer et al., 2017). Specifically, endohyphal bacteria not only promote fungal growth and reproduction through metabolic interactions (Mondo et al., 2017), as exemplified by *Burkholderia* enhancing spore germination and hyphal growth in *Rhizopus* fungi (Ohshima et al., 2016), but also endow their hosts with novel ecological functions (Giger et al., 2020). Furthermore, endohyphal bacteria can enhance the environmental adaptability of their hosts through the synthesis of bioactive metabolites (Büttner et al., 2021) and improve host disease resistance by producing diverse secondary metabolites (Schoonbeek et al., 2007). Collectively, these findings establish the fungal-endohyphal bacterial symbiotic system as a valuable model for studying microbial interactions, evolutionary adaptation, and metabolic regulation, particularly for understanding how microorganisms collaboratively respond to environmental stresses (Deveau et al., 2018).

In general, fungal abundance tends to decline under extremely arid conditions (Xu et al., 2022). However, certain fungi have evolved unique survival strategies to withstand such environmental stresses. These strategies include increasing the proportion of unsaturated fatty acids to enhance membrane fluidity (De Mesquita et al., 2024; Hauschild et al., 2017) and alleviating drought-induced oxidative stress through efficient antioxidant enzyme systems (Zhang et al., 2020; Menezes-Benavente et al., 2004; Apel and Hirt, 2004). More importantly, fungi can modulate their intracellular metabolism, channeling limited carbon sources into triacylglycerols (TAG) biosynthesis. TAGs serve not only as energy reserves but also due to their hydrophobic nature and accumulation process, contribute to osmotic stress mitigation–an essential physiological response for fungal survival under environmental pressure (Hauschild et al., 2017; Breuer et al., 2013). However, research on endohyphal bacteria and their diversity in fungi inhabiting arid environments remains extremely limited.

The Toksun region of Xinjiang, China, represents an extremely arid zone characterized by prolonged exposure to high temperature, salinity, and drought stress (Shao et al., 2024). Its unique geographical and climatic conditions have shaped distinctive and resilient microbial communities. Recently, our team isolated and excavated some oil-producing fungi from the arid environment of Toksun, Xinjiang (Feng et al., 2025), and found that some of these oil-producing fungi harbor endohyphal bacteria. However, previous studies have suggested that symbiotic interactions are key determinants of the physiology and lipid accumulation in oil-producing microorganisms (Deka et al., 2020). Endohyphal bacteria can significantly affect the physiological state and metabolic profiles of their fungal hosts. For instance, by modulating the activity of key enzymes such as diacylglycerol kinase (DGK), endohyphal bacteria can redirect lipid metabolic fluxes and alter fatty acid composition, thereby influencing both the symbiotic relationship and the environmental adaptability of the host fungus (Lastovetsky et al., 2016). Nevertheless, the distribution of oil-producing fungal species in this arid environment, the presence of endohyphal bacteria associated with these fungi, as well as the community structure and diversity of these microorganisms, remain poorly understood and warrant further in-depth investigation.

Consequently, this study aims to conduct a preliminary analysis of the community structure and diversity characteristics of oil-producing fungi and their endohyphal bacteria in this habitat, thereby providing a theoretical foundation for understanding the fungal-bacterial symbiotic system in extremely arid environments and laying the groundwork for future studies on their ecological functions and the development of related microbial resources.

## 2 Materials and methods

### 2.1 Source of soil samples

Soil samples were collected in June 2023 from Toksun County (88.465833°E, 42.329167°N), Turpan City, Xinjiang, China. Using a random sampling method, five representative arid ecological zones in the region were selected for sampling. Surface soil samples (0–20 cm depth) were collected, and visible stones were removed. The soil samples were thoroughly homogenized, and approximately 2 kg of composite soil was retained and transported to the laboratory, where it was stored at 4 °C for subsequent experiments (Li et al., 2025). The basic physicochemical properties of the experimental soil were as follows: water content of 0.22%, pH 9.08, total soluble salt content of 668.4 g/kg, organic matter content of 7.9 g/kg, total nitrogen content of 0.13 g/kg, available nitrogen concentration of 1.1 mg/kg, and available potassium concentration of 239.2 mg/kg.

### 2.2 Isolation and screening of oil-producing fungi in Toksun arid environments

Weigh 5 g of the soil sample into 45 mL of sterile water, and the mixture was shaken at 30 °C and 150 rpm for 30 min. The suspensions were serially diluted to 10^−3^. Then, 100 μL of the 10^−1^, 10^−2^, and 10^−3^ dilutions were spread onto potato dextrose agar (PDA; 6 g/L potato extract, 20 g/L dextrose, and 20 g/L agar) plates, incubated at 30 °C for approximately 7 days (Yao, 2017). Distinct colonies were selected for purification and inoculated onto PDA slants for further use.

The isolated fungal strains were inoculated onto PDA plates, into which sterile cover slips were inserted at an angle. After hyphal growth covered the cover slips, the slips were carefully removed and stained with Sudan Black B (Macklin Biochemical, Shanghai, China) for 20–30 min. The excess stain was removed using xylene(Tianjin Zhiyuan Chemical Products, Tianjin, China), and the slips were air-dried and counterstained with safranin solution for 1–2 min. Subsequently, the excess dye was rinsed off with sterile water. After air-drying, the samples were examined under a microscope to screen for oil-producing fungi. Among them, the hyphae were red, and while lipid granules within the fungal strains were stained dark blue-black (He, 2024).

### 2.3 Molecular identification of oil-producing fungi

The oil-producing fungi isolated were first surface-sterilized with 70% ethanol for 2 min, followed by five rinses with sterile water to eliminate external contaminants (Cheng et al., 2022). Total genomic DNA was extracted from the fungal isolates using a Fungal Genomic DNA Extraction Kit (Sangon Biotech, Shanghai, China). After extraction, the DNA concentration was quantified using a Qubit fluorometer (Zheng et al., 2023), with the quantitative data presented in Supplementary Table S1. Universal primers ITS1 (5’-TCCGTAGGTGAACCTGCGG-3′) and ITS4 (5’-TCCTCCGCTTATTGATATGC-3′) were used for PCR amplification (Xie et al., 2024). Each 30 μL PCR reaction mixture contained 25 μL of 2 × PCR Master Mix, 1 μL of each primer (10 μmol/L), 2 μL of DNA template (20 ng/μL), and nuclease-free water to volume. PCR amplification was performed under the following conditions: initial denaturation at 95 °C for 5 min; followed by 30 cycles of 94 °C for 45 s, 50 °C for 30 s, and 72 °C for 45 s; with a final extension at 72 °C for 1 min (Feng et al., 2025). The amplification products were verified by 1% agarose gel electrophoresis, which yielded a single distinct band of approximately 600 bp, confirming amplification specificity. The PCR amplification profile is presented in Supplementary Figure S1. The purified PCR products were subjected to Sanger sequencing (Novogene Bioinformatics Technology Co., Ltd.). The raw sequences were assembled, manually checked, and subjected to homology analysis using the BLAST database. A phylogenetic tree was subsequently constructed using the Neighbor-Joining method to determine the taxonomic position of the strains. The final sequence data have been deposited in the GenBank database under accession numbers PX443408 to PX443423.

### 2.4 SYTO 9 staining detection of endohyphal bacteria in oil-producing fungi

The nucleic acid-specific fluorescent dye SYTO 9 was used to detect the endohyphal bacteria within oil-producing fungi. This dye penetrates bacterial cell membranes and specifically binds to DNA, producing bright green fluorescence upon excitation (McGoverin et al., 2020). The procedure was as follows: the oil-producing fungi were cultured on PDA plates for 4 days. Subsequently, 15 μL of SYTO 9 dye (Kanglang Biotechnology, Shanghai, China) was added to the slide, after which a coverslip containing fungal hyphae was placed on the slide, and incubated in the dark at room temperature for 15–20 min. The samples were then observed under confocal laser scanning microscopy (Zeiss, Germany) for rapid detection of endohyphal bacteria (Zheng et al., 2024; Feng, 2023; Hazarika et al., 2020; Bao, 2021; Richter et al., 2023; Richter et al., 2022).

### 2.5 High-throughput sequencing of endohyphal bacteria in oil-producing fungi

Genomic DNA previously extracted in Section 2.3 was diluted with sterile water to a final concentration of 1 ng/μL for subsequent endohyphal bacterial community analysis. To monitor potential contamination and validate experimental reproducibility, negative controls and positive controls were incorporated into the sequencing workflow. The 16S rRNA V5-V7 hypervariable region was PCR-amplified using the following primers: 799F(5’-AACMGGATTAGATACCCKG-3′) and 1193R(5’-ACGTCATCCCCACCTTCC-3′) (Fu et al., 2022; Beckers et al., 2016). Each PCR reaction contained 15 μL of Phusion High-Fidelity PCR Master Mix; 0.2 μM of each primer, and approximately 10 ng template DNA. Thermal cycling consisted of initial denaturation at 98 °C for 1 min, followed by 30 cycles of denaturation at 98 °C for 10 s, annealing at 50 °C for 30 s, and elongation at 72 °C for 30 s and 72 °C for 5 min. The amplification products were verified by electrophoresis with 2% agarose gel. After verification, the PCR products were purified and quantified using enzyme labeling. Equimolar amounts of the PCR products were pooled based on concentration, thoroughly mixed, and subjected to gel electrophoresis again to recover the target bands. Subsequently, the sequencing library was constructed, and the quality of the constructed library was assessed using Qubit and quantitative PCR (qPCR). After quality control, sequencing was performed on the NovaSeq6000 platform (Illumina) by Novogene Bioinformatics Technology Co., Ltd. (Beijing, China) (Chen et al., 2021).

### 2.6 Processing of high-throughput sequencing results

High-throughput sequencing data were processed using a standardized bioinformatics pipeline comprising the following steps: (1) Data demultiplexing: Raw sequence data were demultiplexed and reformatted using Python (v3.6.13); (2) Sequence assembly: Paired-end reads were merged using FLASH (v1.2.11) to generate raw tags (Magoč and Salzberg, 2011); (3) Quality filtering: Raw tags were processed using fastp (v0.23.1) to remove low-quality reads and short fragments, generating high-quality clean tags (Bokulich et al., 2012); (4) Chimera removal: Potential chimeric sequences were identified by comparing tags against the SILVA 16S/18S database using vsearch (v2.16.0) (Edgar et al., 2011) and subsequently removed to obtain effective tags. (5) Contamination filtering: To address potential contamination from laboratory contaminants, we employed the R package decontam (v1.12.0) for systematic removal of contaminant sequences. Specifically, amplicon sequence variants (ASVs) that were significantly more abundant in the negative control samples compared to the experimental samples were classified as potential contaminants and subsequently filtered out. (6) Denoising and ASV inference: Effective tags were denoised and used to infer ASVs with the DADA2 algorithm implemented in the QIIME 2 framework (v2022.02) (Zheng et al., 2023; Zheng et al., 2024; Bokulich et al., 2018). Sequencing depth was normalized across all samples by rarefaction prior to diversity analyses to ensure comparability. Representative ASVs were taxonomically annotated to determine endohyphal bacterial species composition, distribution, and diversity with oil-producing fungi. Functional prediction of endohyphal bacterial communities was conducted using PICRUST2.

### 2.7 Data processing

All statistical analyses in this study were conducted with a significance threshold of *p* < 0.05. Phylogenetic trees of oil-producing fungi were constructed based on ITS sequences using the neighbor-joining method implemented in MEGA 11. Venn diagrams depicting community composition, petal plots, and species abundance heatmaps for endohyphal bacterial communities were generated via the Novomagic cloud computing platform.^1^ Furthermore, the Kruskal-Wallis test was also applied to assess significant differences in community structure across fungal genera based on the Bray–Curtis dissimilarity matrix.

## 3 Results and analysis

### 3.1 Screening of oil-producing fungi in arid environments and endohyphal bacteria staining

**Figure 1:**
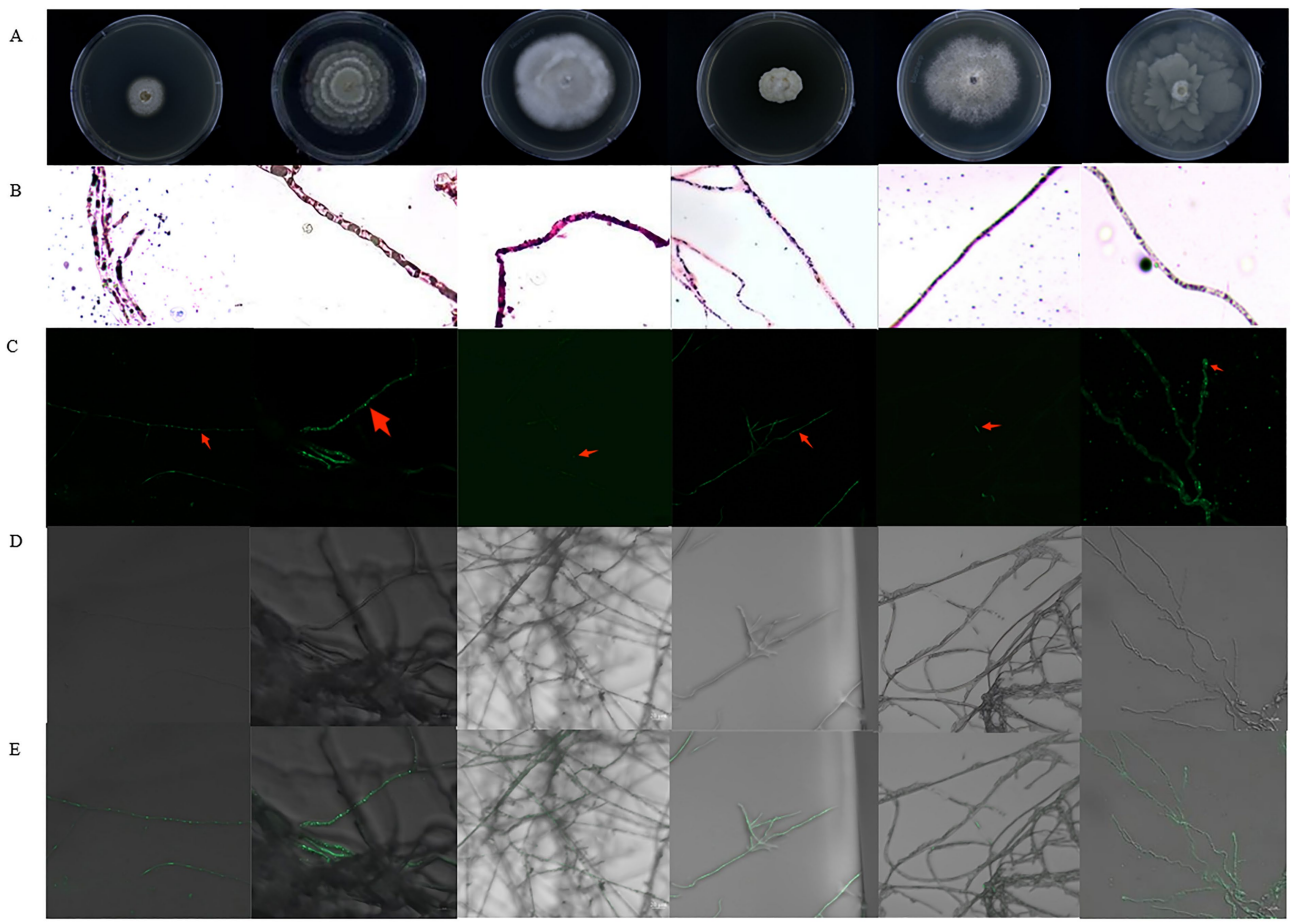
Part of oil-producing fungal colony and hyphae staining. **(A)** Morphological characteristics of oil-producing fungal colonies. **(B)** Sudan Black B staining method for detecting oil-producing fungi. **(C–E)** Confocal laser scanning microscopy images showing endohyphal bacteria within hyphae: **(C)** Fluorescence signal from SYTO 9 staining, highlighting bacterial DNA localization; **(D)** Bright-field image corresponding to the hyphal structure; **(E)** Overlay of fluorescence and bright-field channels, confirming intracellular bacterial presence. Scale bars: 20 μm.

**Figure 2:**
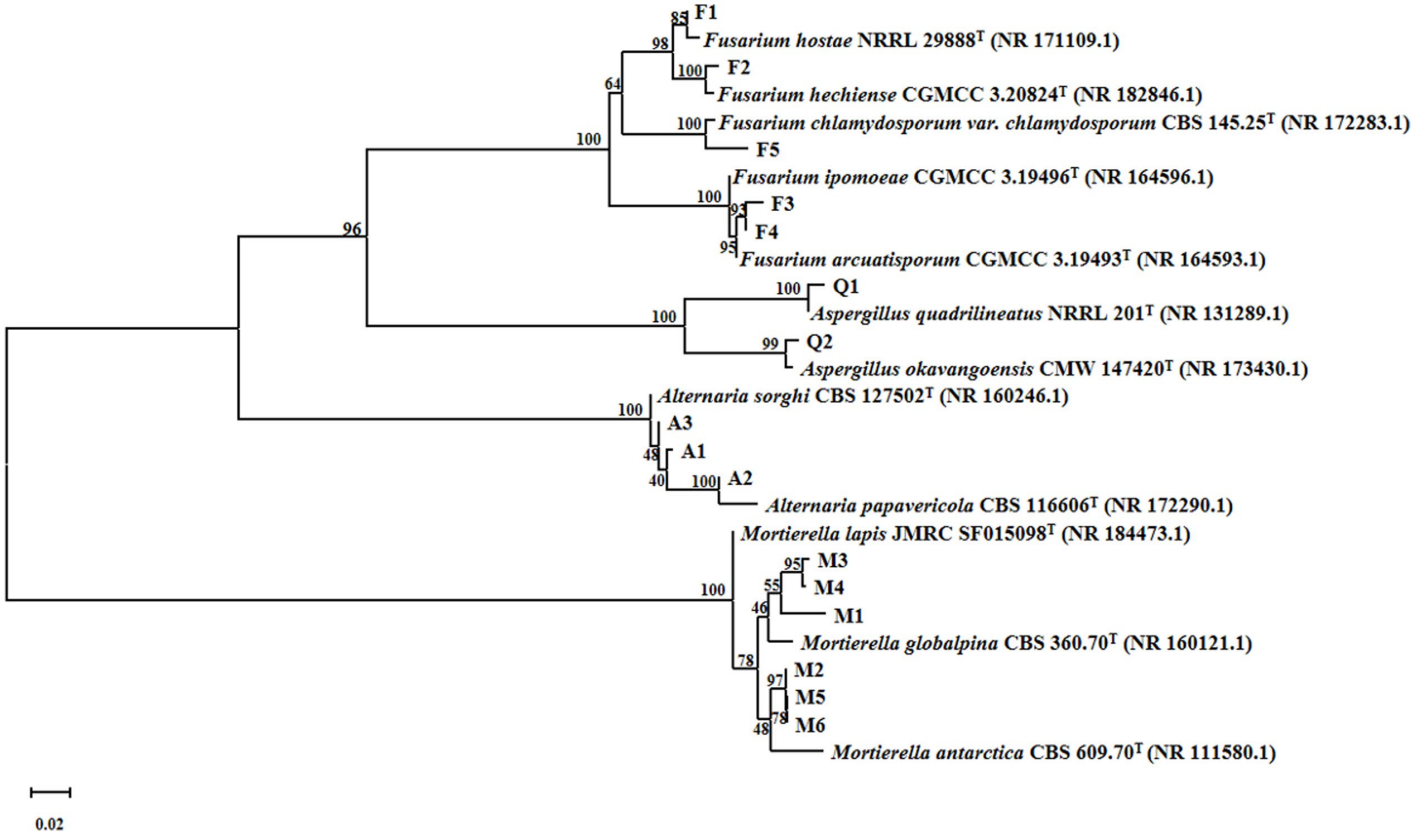
The phylogenetic tree of oil-producing fungi containing endohyphal bacteria was constructed by using the ITS gene sequence and the neighbor-joining method.

A total of 237 fungal strains were isolated, of which 26 demonstrated notable oil-producing capacity as determined by Sudan Black B staining. SYTO 9 fluorescence staining further revealed that 16 of these strains harbored endohyphal bacteria, representing 61.5% of the oil-producing fungi examined (Figure 1). ITS sequencing identified these 16 fungal strains belonging to four genera-*Mortierella*, *Aspergillus*, *Fusarium*, and *Alternaria*-encompassing a total of 10 species. Among them, *Mortierella* species were predominant, accounting for 37.6% of the identified oil-producing fungi (Figure 2).

### 3.2 Analysis of the species composition and diversity of endohyphal bacteria in oil-producing fungi

High-throughput sequencing was applied to characterize the endohyphal bacterial communities associated with oil-producing fungi. A total of 1,543,950 raw sequences were obtained across all samples. After stringent bioinformatic filtering, including the removal of low-quality reads and chimeric sequences, 763,395 high-quality sequences were retained for downstream analyses. The rarefaction curve reached a clear saturation plateau (Supplementary Figure S2), indicating that the sequencing depth was sufficient to capture the majority of the bacterial diversity. Sequence data were processed using the DADA2 algorithm to infer exact amplicon sequence variants (ASVs), yielding a total of 63 ASVs across the dataset. The taxonomic composition of the endohyphal bacterial communities was subsequently examined at both the phylum and genus levels.

#### 3.2.1 Diversity analysis of endohyphal bacteria in oil-producing fungi

**Figure 3:**
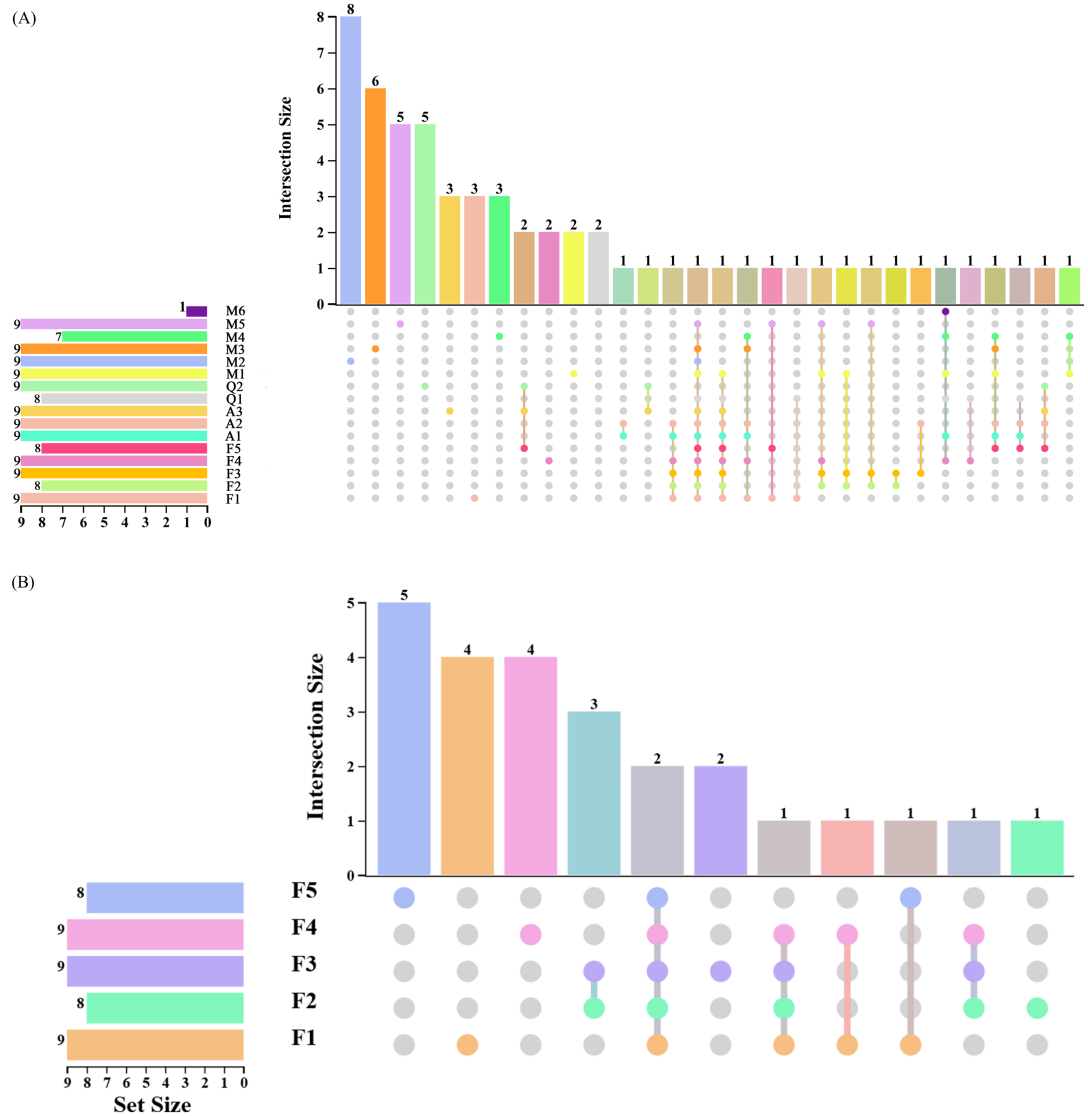
Distribution of ASVs across four fungal genera, including a detailed breakdown of ASV distribution within the genus *Fusarium*. **(A)** UpSet plot illustrating the distribution of ASVs among the genera *Fusarium*, *Mortierella*, *Aspergillus*, and *Alternaria*. Vertical bars in the lower-left panel represent the total number of ASVs detected in each genus, while the bar plot and connecting lines above indicate shared ASVs within and across genera. **(B)** UpSet plot showing the distribution of ASVs among the five *Fusarium* species, highlighting differences in endohyphal bacterial community composition among species.

Sequencing results showed that endohyphal bacterial ASVs were assigned to 6 phyla and 35 genera, indicating substantial taxonomic diversity. Among the 16 oil-producing fungi, most harbored 8–9 endohyphal bacterial ASVs, whereas only a single AVS was detected in strain M6. No ASVs were shared across all samples (Figure 3A), highlighting pronounced heterogeneity in endohyphal bacterial communities among fungal genera. Notably, two ASVs were consistently shared among all five *Fusarium* strains, whereas similar patterns of ASV sharing were less evident in other fungal genera (Figure 3B). These findings indicate that variation in endohyphal bacterial composition occurs not only between different species within a given fungal genus but also among strains of the same species.

#### 3.2.2 Species composition analysis of endohyphal bacteria in oil-producing fungi

**Figure 4:**
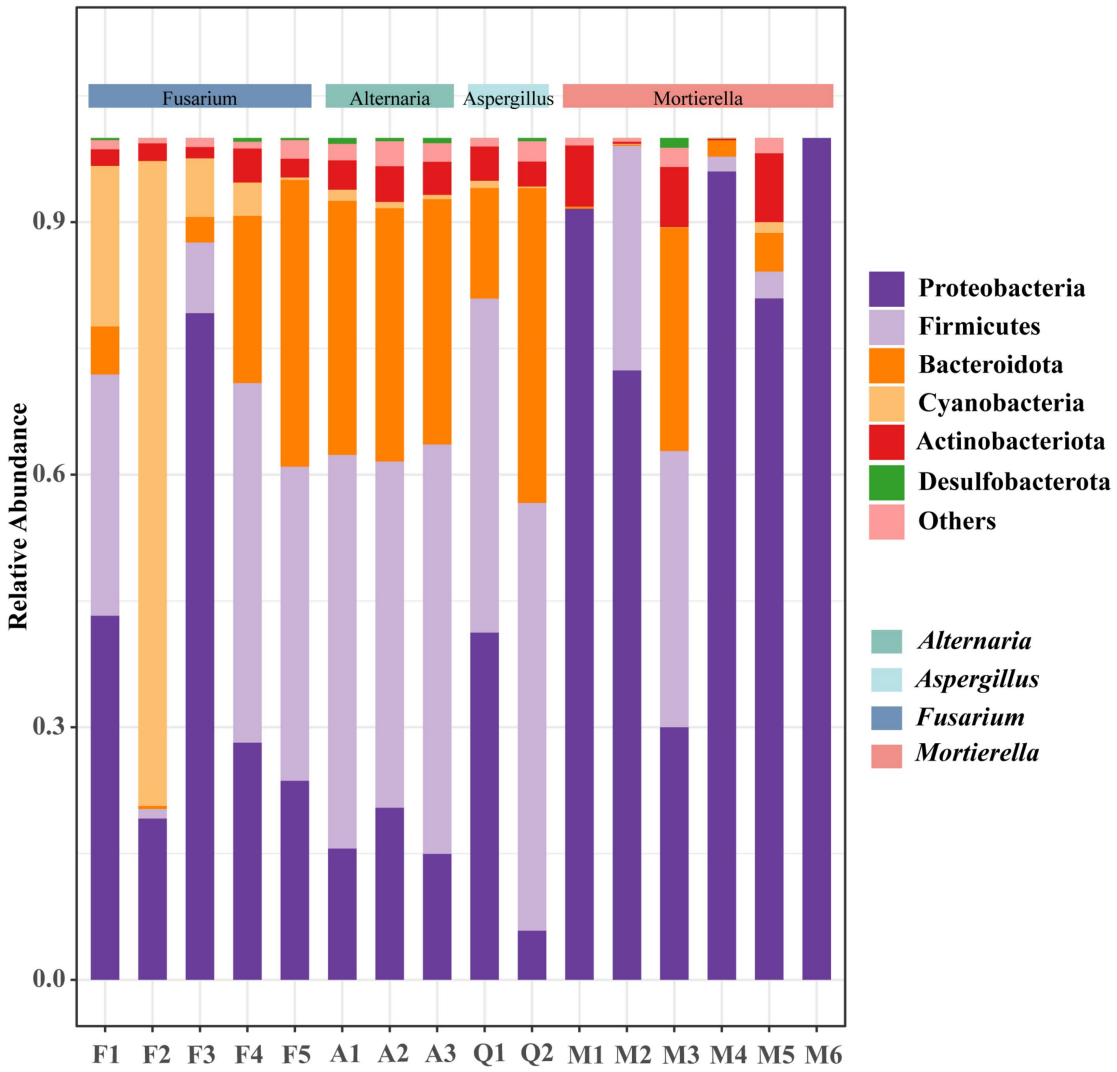
Distribution map of species composition at the level of endohyphal bacteria phylum of oil-producing fungi.

**Figure 5:**
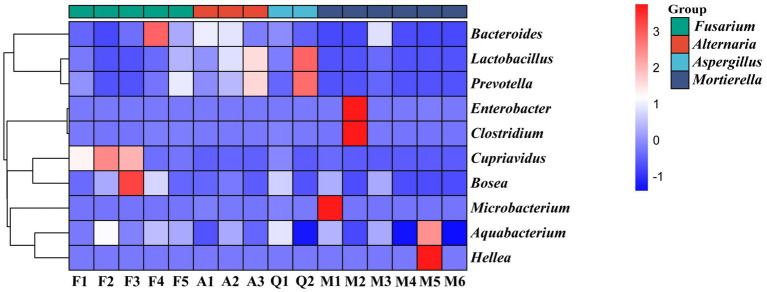
Correlation heat map of dominant genera of endohyphal bacteria of different species of oil-producing fungi.

The composition and distribution of endohyphal bacterial communities were shown in Figures 4, 5. At the phylum level, endohyphal bacteria were assigned to six phyla, predominantly Firmicutes (1.1–50.8%), Bacteroidota (0.2–37.4%), Proteobacteria (5.8–100%), and Actinobacteria (0.2–8.2%) (Figure 4). Marked compositional differences were observed among fungal genera. In *Alternaria* strains, Firmicutes (41.1–48.6%) and Bacteroidota (29.1–30.2%) dominated the community; however, inter-strain variation within this genus was minimal. In contrast, *Mortierella* strains exhibited pronounced strain-level specificity, with Proteobacteria (30–100%) as the dominant phylum, followed by Firmicutes (1.8–32.8%). Notably, strain M3 exhibited higher microbial diversity and a community structure that differed substantially from those of other *Mortierella* strains. Conversely, strain M6 displayed an extremely simplified community structure, composed exclusively of Proteobacteria (100%). Collectively, these findings provide preliminary insights into the phylum-level structure of endohyphal bacterial communities across diverse fungal hosts.

At the genus level, endohyphal bacterial communities exhibited pronounced variation across fungal hosts (Figure 5). In *Mortierella* strains, the core microbiota was dominated by *Enterobacter* (56.2%), followed by *Clostridium* (25.4%), and *Hellea* (12.1%). However, notable strain-level differences in dominant taxa were observed. Strain M1 was enriched in *Microbacterium* (6.7%), whereas M2 contained higher proportions of *Enterobacter* (56.2%) and *Clostridium* (25.4%). In contrast, M5 was characterized by elevated abundances of *Hellea* (12.1%) and *Aquabacterium* (3.9%). In comparison, *Fusarium* strains possessed distinct community structures dominated by *Cupriavidus*. Specifically, *Cupriavidus* constituted 64.4% of the endohyphal bacterial community in strain F3, with *Bosea* (3.2%) present at lower abundance, whereas F4 was characterized by a higher proportion of *Bacteroides* (17.4%). Overall, endohyphal bacterial communities exhibited marked heterogeneity at both the genus level and among strains within the same genus, providing testable hypotheses for future studies aimed at elucidating the mechanisms underlying fungal-bacterial interactions and the origins of strain-specific differences.

#### 3.2.3 Beta diversity analysis of endohyphal bacteria in oil-producing fungi

**Figure 6:**
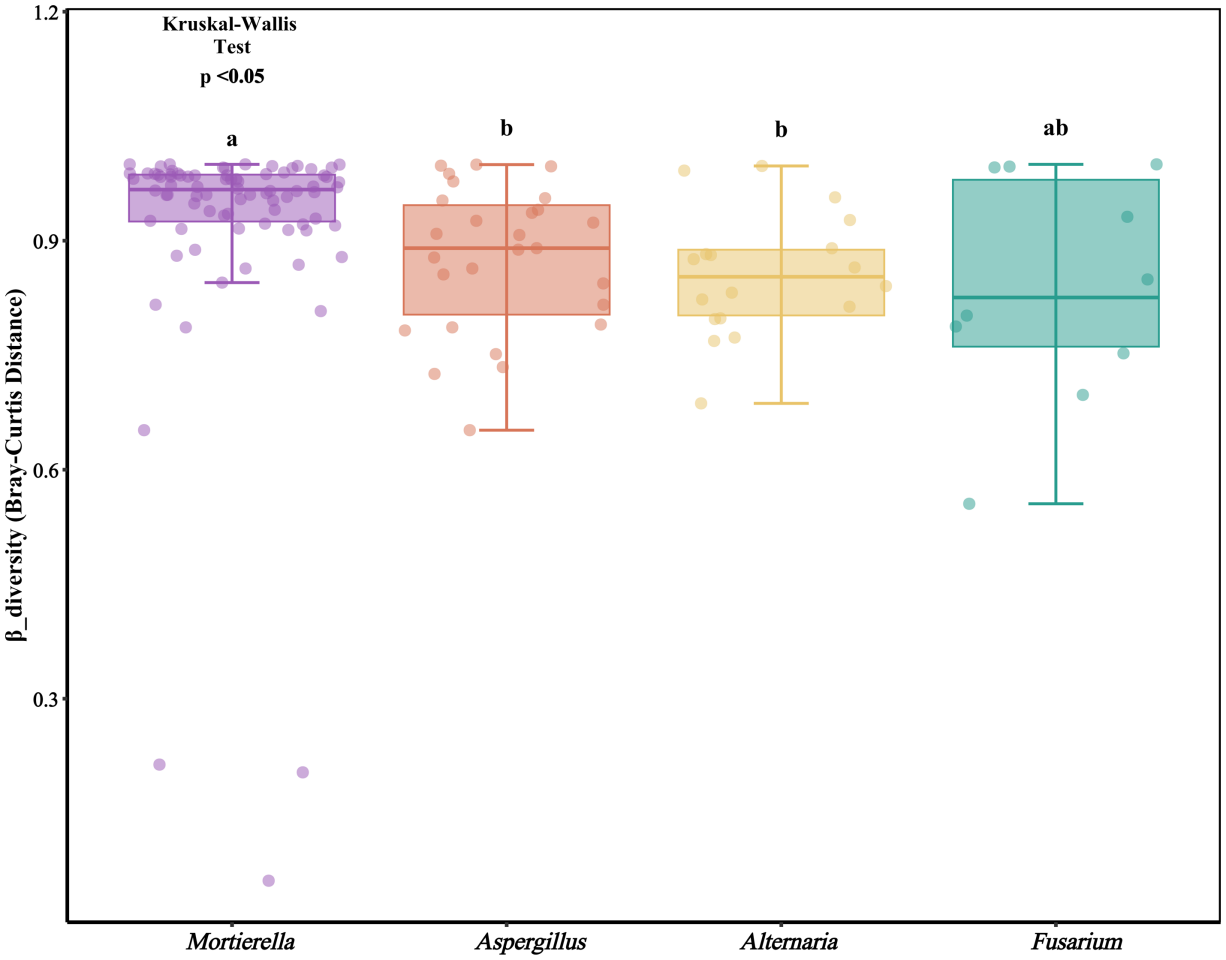
Statistical comparison of community dissimilarities (Bray-Curtis distance) among groups using the Kruskal-Wallis test (*p* < 0.05).

The results of Principal Component Analysis (PCA) revealed significant differences in *β*-diversity among endohyphal bacterial communities of oil-producing fungi across different genera (Figure 6). To statistically evaluate compositional differences among oil-producing fungal genera, an analysis of variance based on Bray–Curtis dissimilarity was performed. The results revealed a statistically significant effect of fungal genus on community structure (*p* < 0.05). Specifically, the endohyphal bacterial community associated with *Mortierella* significantly diverged from those of *Alternaria* and *Aspergillus*, whereas no significant difference was detected between *Alternaria* and *Aspergillus*, indicating high similarity in their endohyphal bacterial communities. Moreover, the endohyphal bacterial communities of *Fusarium* strains exhibited considerable heterogeneity: certain strains displayed marked distinctions from other genera, while others showed partial compositional overlap.

### 3.3 Functional prediction analysis of endohyphal bacteria in oil-producing fungi

**Figure 7:**
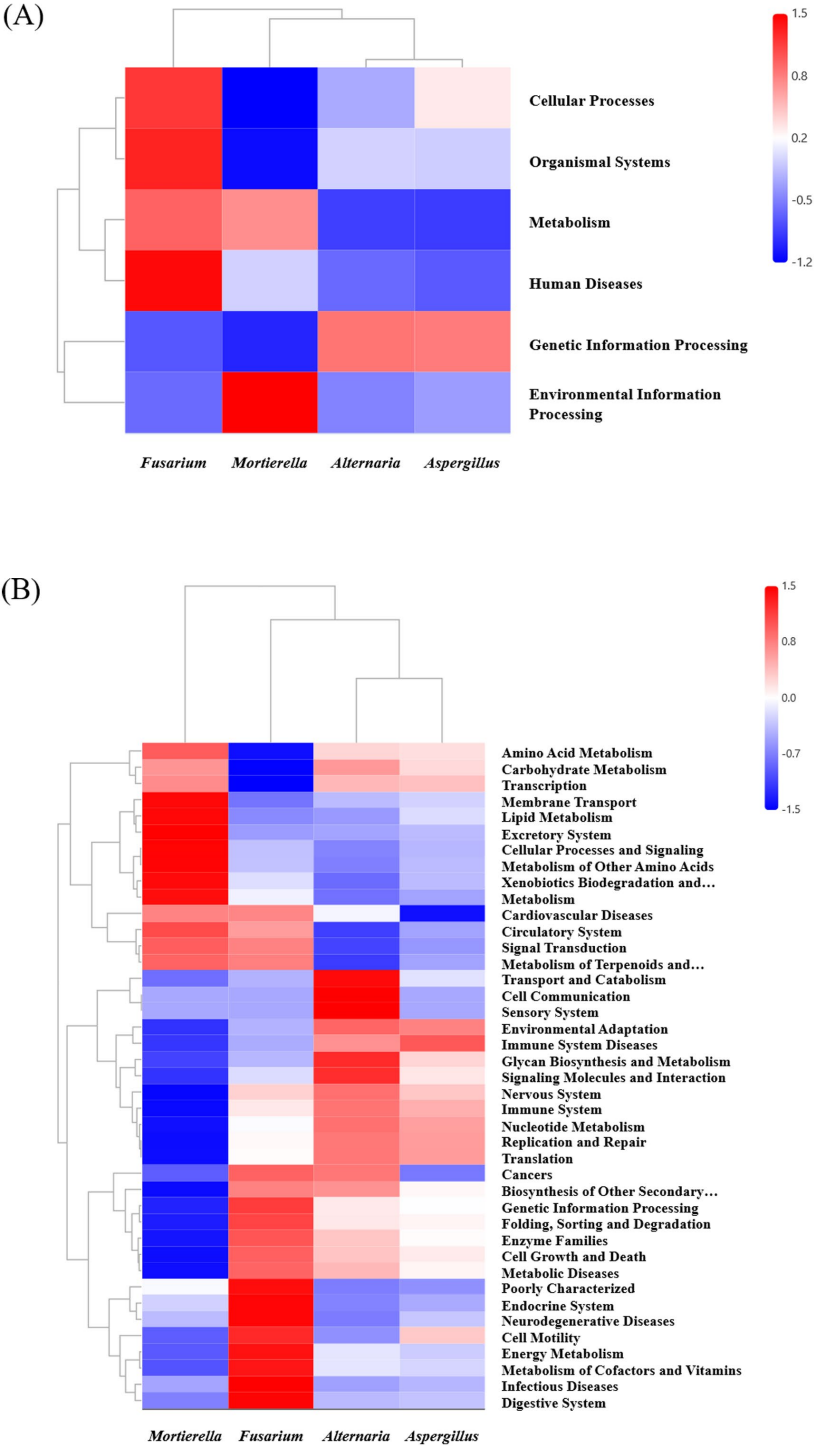
Predictive functional profiling of endohyphal bacterial communities associated with oil-producing fungi based on PICRUSt2 analysis. **(A)** Heatmap showing the predicted relative abundances of KEGG Level 1 functional categories across fungal genera. **(B)** Heatmap showing the predicted distribution of KEGG Level 2 functional categories.

PICRUSt2 functional prediction provided an initial overview of the potential functional profiles of endohyphal bacterial communities associated with oil-producing fungi. At the KEGG Level 1 classification, predicted functions were mainly distributed among metabolism, genetic information processing, environmental information processing, cellular processes, and human-disease-associated categories. With relative abundances of 47.4–63.7%, 14.5–33.6%, 14.2–19.8%, 3.1–3.9%, and 0.134–0.138%. Predicted functional profiles varied across fungal genera (Figure 7A). For example, endohyphal bacteria associated with *Fusarium* were predicted to contribute mainly to metabolism (63.7%) and cellular processes (3.9%), whereas those associated with *Alternaria* and *Aspergillus* showed relatively higher representation in genetic information processing (33.3–33.6%). In contrast, endohyphal bacterial communities in *Mortierella* were predominantly associated with metabolic functions (61.8%) and environmental information processing (19.8%).

At the KEGG Level 2 category, the predicted functions also varied among fungal genera (Figure 7B). *Fusarium*-associated endohyphal bacteria were predicted to contribute more strongly to energy metabolism (7.1%) and cofactor and vitamin metabolism (4.8%). *Mortierella*-associated bacteria showed greater predicted representation in carbohydrate (10.3%), amino acid (9.9%), lipid metabolism (3.5%), as well as membrane transport (16.2%). *Alternaria*-associated bacteria showed relatively higher contributions to predicted transport and carbohydrate pathways (10.3%), as well as cellular communication (4.4%) and signal transduction (3.1%). *Aspergillus*-associated bacteria were predicted to contribute more to pathways related to environmental adaptation (4.5%) and translation (5.1%). Importantly, these inferences are derived solely from PICRUSt2 computational predictions based on 16S rRNA gene data, and their biological relevance requires validation through culture-based, genomic, or transcriptomic approaches.

## 4 Discussion

The symbiotic interactions between fungi and endohyphal bacteria represent an emerging research frontier in microbial ecology. However, their distribution patterns and ecological functions in arid environments remain poorly characterized. In this study, we conducted a preliminary analysis of 26 oil-producing fungal strains, isolated from an extremely arid habitat in Xinjiang characterized by high alkalinity (pH 9.08), elevated salinity (668.4 g/kg total salts), and very low water content (0.22%). We found that 61.5% (16 strains) of the fungal isolates harbored detectable endohyphal bacterial communities, respresenting four genera. This finding indicates that fungal-bacterial symbioses can persist even under multiple extreme abiotic stresses. Although the colonization rate observed here was lower than that reported by Shaffer et al. (2016) (approximately 75%) and Robinson et al. (2021) (up to 88%) across broader fungal taxa, this difference may reflect the extreme environmental conditions of the host habitat, where high salinity and aridity impose environmental pressures on the establishment and persistence of fungal-bacterial symbioses. Additionally, variation in colonization frequency may also be influenced by host-specific traits or by the limited sample size in our study. Nevertheless, our findings support the growing evidence that endohyphal bacteria are associated with fungi in arid environments and provide new perspectives on the environmental adaptability of fungal–bacterial symbioses.

In terms of community composition, this study elucidates the multi-level distribution characteristics of endohyphal bacteria derived from oil-producing fungi across four genera in arid regions. High-throughput sequencing revealed that Proteobacteria, Firmicutes, and Actinobacteria constituted the dominant phyla of endohyphal bacteria in oil-producing fungi from arid environments. This compositional feature exhibits a high degree of consistency with the findings of Robinson et al. (2021) in fungi from different habitats, further substantiating the universal adaptability of these bacterial phyla to fungal endohyphal environments (Hassani et al., 2018). Notably, distinct host-specific distribution patterns were observed. For instance, in *Mortierella* fungi, Proteobacteria predominates with a relative abundance ranging from 30% to 100%. Among these, *Enterobacter* has been confirmed to colonize *Fusarium* fungi and participate in regulating host sporulation and pathogenicity (Obasa et al., 2019). However, the interaction mechanisms between most bacterial genera and their fungal hosts remain unclear and warrant further investigation. More intriguingly, we identified significant compositional variations among different species within the same genus. For example, strain M6 exhibited an extremely simplified community structure, while strain M3 demonstrated higher microbial diversity. This finding aligns with recent studies, indicating that significant intra-species variations may exist in the composition and presence of endohyphal bacteria (Wallner et al., 2025).

The observed community structural patterns may be influenced by intrinsic biological characteristics of the associated bacterial taxa (Hassani et al., 2018; Wallner et al., 2025). Previous studies have reported that dominant endohyphal bacterial taxa often encode numerous secretion system genes (e.g., Type III secretion systems) (Baltrus et al., 2017), which may enable the delivery of effector proteins into host cells, and modulate host physiological processes (Lackner et al., 2011; Lackner et al., 2010; Deng et al., 2017; Lackner et al., 2011; Miyauchi et al., 2020; Benoit et al., 2014; Kayla et al., 2016). Concurrently, they possess metabolic traits such as diverse carbon utilization pathways, potential nitrogen fixation (Liang et al., 2023), and phytohormone biosynthesis capabilities (Hoffman et al., 2013). Based on these characteristics, we hypothesize that endohyphal bacteria may maintain metabolic activity within the fungal microenvironment and potentially provide conditional metabolic support to their host (Wei et al., 2024; Alabid et al., 2018; Shao et al., 2020). If validated, such mechanisms could contribute to enhancing host environmental adaptability under drought stress.

Functional prediction analysis provides preliminary indications of the potential ecological roles of these associations. Endohyphal bacteria associated with *Fusarium* were predicted to contribute more strongly to energy metabolism and cofactor and vitamin metabolism, a pattern that parallels reported metabolic interactions between *Burkholderia*-*Rhizopus* (Ohshima et al., 2016; Giger et al., 2020). We therefore hypothesize that, under drought stress, such bacteria may support their fungal hosts by contributing to energy homeostasis (Wei et al., 2024) and redox balance (Sun et al., 2023) through analogous metabolic interactions (Gohar et al., 2025). However, these interpretations remain preliminary, as they are inferred solely from genomic predictions and currently lack direct experimental validation. For *Mortierella*-associated endohyphal bacteria, predicted functions were more strongly represented in carbohydrate, amino acid, and lipid metabolism. These patterns speculate a possible role in influencing host metabolic activity, although their contributions to host lipid production and biological control remain hypothetical (Büttner et al., 2021; Lastovetsky et al., 2016; Wei et al., 2024; Thomma, 2021). Nonetheless, such proposed mechanisms require confirmation through targeted experimental studies. For *Alternaria* hosts, endohyphal bacterial functional predictions showed greater representation of signal transduction and cell communication pathways. While previous studies have suggested that collective bacterial behaviors may contribute to the maintenance of symbiotic relationships (Kai et al., 2012), it remains speculative and requires validation through molecular approaches. Meanwhile, the higher predicted representation of replication-repair and environmental adaptation pathways in *Aspergillus*-associated endohyphal bacteria may indicate potential mechanisms related to genomic stability and stress-responsive regulation. This pattern is consistent with studies showing that bacteria such as *Pseudomonas* can enhance host stress resistance through secondary metabolism, illustrating the diverse predicted pathways that may contribute to host environmental adaptability (Schoonbeek et al., 2007).

Based on the community structure and functional prediction data obtained in this study, we propose a preliminary hypothesis that, under drought stress, oil-producing fungi may engage in a putative interactive system with functionally diverse endohyphal bacteria. Such interactions could contribute to ecological adaptability in extreme environments through the hypothesized metabolic synergy and functional complementarity. This hypothesis provides a new perspective for understanding the environmental resilience of microbial symbioses; however, the underlying mechanisms and functional consequences remain to be validated through integrated approaches such as co-culture assays, transcriptomics, and metabolomics.

While this study provides a preliminary assessment of endohyphal bacteria diversity in oil-producing fungi from arid environments, we acknowledge several limitations: Firstly, the limited sample size prevents our ability to draw broad ecological conclusions. Although variations in endohyphal bacterial communities were observed among fungal species, these findings remain preliminary and lack sufficient evidence to support the widespread occurrence or preferential colonization of endohyphal bacteria in oil-producing fungi. Secondly, because of the uneven species-level sample distribution, analyses were primarily conducted at the genus level to provide an initial framework of community structure. Future studies should expand sample size to enable higher-resolution species-level analyses and better clarify the specific interactions between hosts and their associated endohyphal bacteria. In addition, current functional inferences are based solely on bioinformatics predictions; thus, future studies should validate these putative functions through isolation, cultivation techniques, and targeted molecular assays (Baltrus et al., 2018). In conclusion, despite these limitations, the present work provides a basis for future investigations and opens new avenues for exploring the complexity of microbial symbiotic networks in arid environmental ecosystems.

## 5 Conclusion

This study presents the first systematic investigation into the community structure and distribution patterns of endohyphal bacteria associated with oil-producing fungi in the arid region of Toksun, Xinjiang. The principal findings are as follows: (1) Oil-producing fungi in arid habitats harbor endohyphal bacteria, with a colonization rate of 61.5%; (2) The endohyphal bacterial communities were dominated primarily by Proteobacteria and Firmicutes; (3) A clear hierarchical distribution pattern was observed, characterized by both genus-specific differences (e.g., between *Mortierella* and *Fusarium*) and strain-level variations (e.g., between strains M3 and M6). Collectively, these findings provide a preliminary understanding of the compositional structure and distribution patterns of endohyphal bacterial communities across diverse fungal hosts. They establish a foundation for elucidating fungal-bacterial cross-kingdom interactions in arid environments and support future in-depth investigations into their metabolic interaction mechanisms.

## Data Availability

The data presented in this study are publicly available. The final sequencing data is available in the GenBank repository (https://www.ncbi.nlm.nih.gov/genbank), under accession numbers PX443408 to PX443423.

## Footnotes

1
magic.novogene.com
