# Supplementary material for: Diversity analysis of endohyphal bacteria in oil-producing fungi inhabiting arid environments
Source: Front Microbiol. 2026 Jan 6;16:1712713. doi: 10.3389/fmicb.2025.1712713 (PMC12815796; doi:10.3389/fmicb.2025.1712713)
Supplement: Supplementary file 1 [file Data_Sheet_1.PDF]

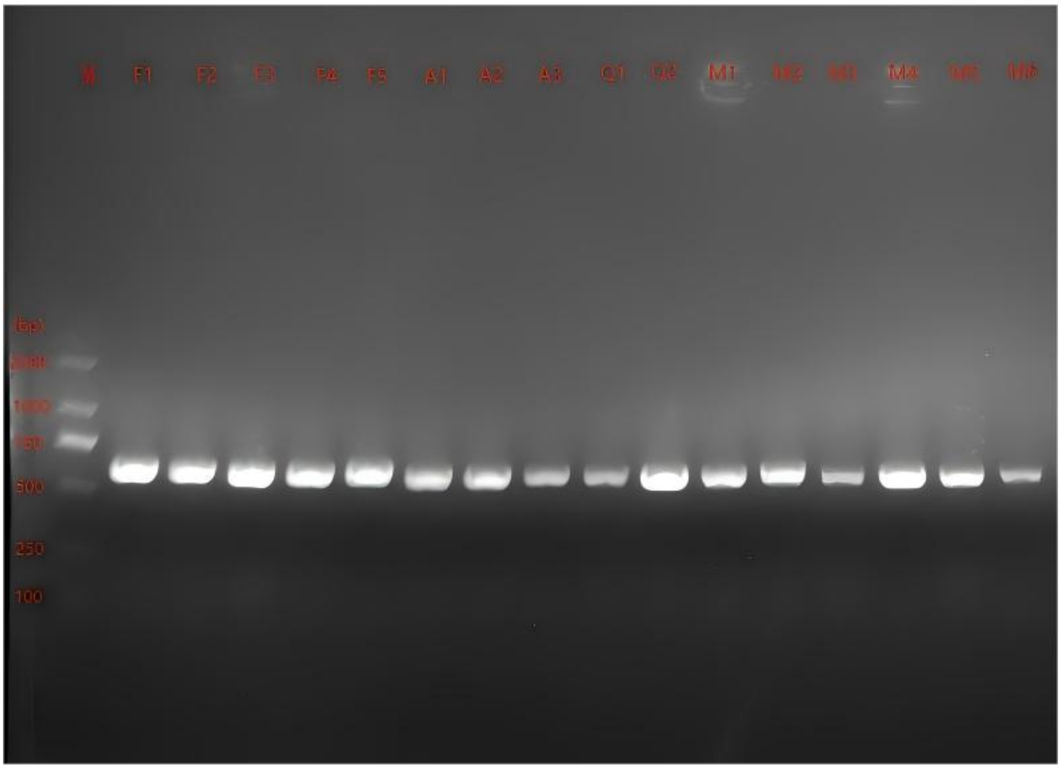


Figure S1. Agarose gel electrophoresis results of fungal ITS amplification fragments.

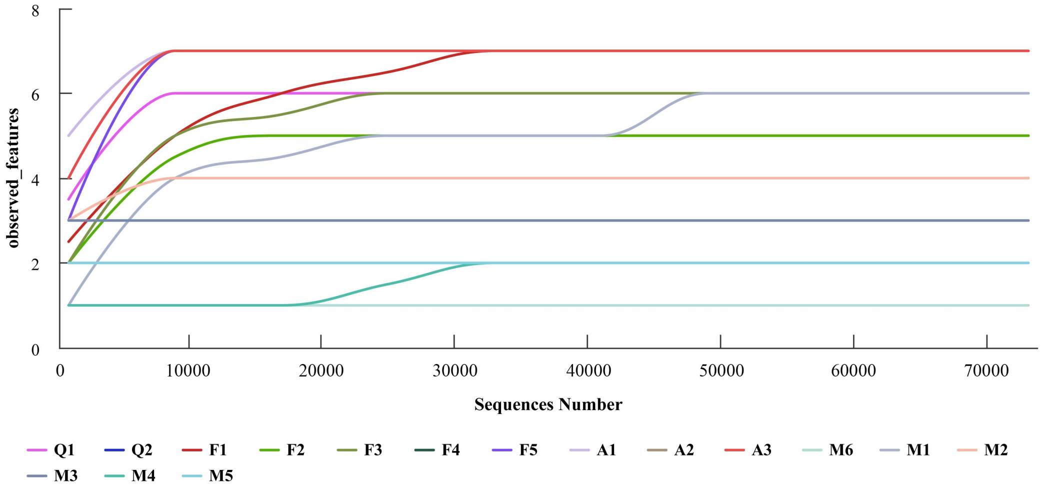


Figure S2. Rarefaction curves of endohyphal bacterial communities associated with oil-producing fungi.

Table S1. DNA concentration of oil-producing fungi.

| Sample Name | Concentration (ng/μL) | Volume (μL) | Total Amount (ng) |
|---|---|---|---|
| F1 | 14.39 | 30 | 431.7 |
| F2 | 17.5 | 30 | 525 |
| F3 | 15.13 | 30 | 453.9 |
| F4 | 17.67 | 30 | 530.1 |
| F5 | 17.39 | 30 | 521.7 |
| A1 | 14.81 | 30 | 444.3 |
| A2 | 20.15 | 30 | 604.5 |
| A3 | 17.54 | 30 | 526.2 |
| Q1 | 15.51 | 30 | 465.3 |
| Q2 | 19.93 | 30 | 597.9 |
| M1 | 15.1 | 30 | 453 |
| M2 | 16.76 | 30 | 502.8 |
| M3 | 11.77 | 30 | 353.1 |
| M4 | 9.11 | 30 | 273.3 |
| M5 | 19.96 | 30 | 598.8 |
| M6 | 20.29 | 30 | 608.7 |
